# Perioperative Fluid Management in Paediatric Liver Transplantation: A Systematic Review

**DOI:** 10.4274/TJAR.2024.241564

**Published:** 2024-07-12

**Authors:** Raihanita Zahra, Andi Ade Wijaya Ramlan, Christopher Kapuangan, Rahendra Rahendra, Komang Ayu Ferdiana, Arif Hari Martono Marsaban, Aries Perdana, Nathasha Brigitta Selene

**Affiliations:** 1 Universitas Indonesia Faculty of Medicine, Department of Anaesthesiology and Intensive Care, Central Jakarta, Indonesia

**Keywords:** Children, fluid management, liver transplantation, mortality

## Abstract

Perioperative fluid management remains a challenging aspect of paediatric liver transplantation (LT) because of the risk of postoperative complications and haemodynamic instability. Limited research has specifically investigated the impact of fluid management and transfusion on mortality and morbidity in pediatric LT patients. This systematic review summarizes the evidence regarding perioperative fluid management and its clinical outcomes in paediatric LT patients. All primary studies published in English evaluating perioperative fluid management in paediatric LT patients were eligible. PubMed, EBSCOHost, Embase, Proquest, and Google Scholar databases were searched from inception to December 19, 2023. Risks of bias were assessed using the Joanna-Briggs Institute checklist. The results were synthesized narratively. Five retrospective cohort studies of good-excellent quality were included in this review. Two studies evaluated intraoperative fluid administration, one study compared postoperative fluid balance (FB) with outcomes, and two studies compared massive versus non-massive transfusion. A higher mortality rate was associated with intravenous lactated ringer’s (LR) than with normal saline, but not with massive transfusion (MT). Longer hospital stays were correlated with MT, >20% positive FB in the first 72 hours, and greater total intraoperative blood product administration. Higher intraoperative fluid administration was associated with a greater thrombotic risk. Additionally, intraoperative MT and lR infusion were associated with an increased risk of 30-day graft loss and graft dysfunction, respectively. Fluid management may impact the outcomes of paediatric LT recipients. These findings underscore the need for more studies to explore the best fluid management and evaluation strategies for children undergoing LT.

**Main Points**

⦁ Perioperative fluid management may affect the clinical outcomes of paediatric liver transplant recipients, such as mortality rate, hospital length of stay, postoperative complications, and graft health. A higher mortality rate was found in those receiving intravenous lactated ringer compared with normal saline.

⦁ Higher intraoperative fluid administration is associated with longer hospital stays and a higher risk of thrombosis. More studies are required to determine the best fluid management and postoperative monitoring for children undergoing liver transplantation.

## Introduction

Liver transplantation (LT) in children is indicated for patients with end-stage liver disease (acute or chronic), hepatic tumors, genetic metabolic diseases, or viral infections. Paediatric LT increases life expectancy and quality of life.^1, 2^ There is almost no perioperative mortality in paediatric LT patients, and they have excellent long-term survival rates.^3^ Scientific advancements have made LT feasible for infants, with a survival rate of 85% at 1 year post-transplantation.^4^

During surgery, periods of haemodynamic instability may occur with significant blood loss and a risk of a systemic inflammatory response leading to endothelial leakage and the shifting of extravascular fluids.^1^ Perioperative fluid management aims to optimize intravascular volume and ensure adequate tissue perfusion, which may reduce the risk of complications and aid rapid recovery.^5^

After undergoing LT, paediatric patients are admitted to the paediatric intensive care unit (PICU) to receive ongoing resuscitation and close monitoring of their intravascular and haemodynamic status, as well as careful titration of medications.^6^ Posttransplantation complications include vascular complications (hepatic artery and portal vein thrombosis), retransplantation, biliary complications, renal complications [acute kidney injury (AKI)], pulmonary complications, and infections.^4^

Several studies have investigated the risks of patient mortality and morbidity in the intraoperative period; however, there are limited data evaluating such risks in the immediate postoperative period and their effect on patient outcomes.^7, 8^ In adult LT recipients, perioperative variables such as haemodynamic variations and transfusion volume have been associated with the postoperative complications mentioned above.^9, 10^ A high intraoperative fluid volume (>260 mL kg^-1^) during paediatric LT is associated with a longer hospital length of stay (LOS), longer mechanical ventilation days, and increased likelihood of requiring red blood cell (RBC) transfusion during the postoperative period (first 72 hours post-operation).^11^ However, Winters et al.^6^ concluded that during the postoperative period, a positive fluid balance (FB) in paediatric LT recipients in the first 3 days postoperatively is associated with poor in-hospital clinical outcomes. In addition, children who undergo LT may experience haemodynamic derangements in diseased livers and are at increased risk of thrombotic complications and hemorrhage. There are limited data on intraoperative blood loss and its association with postoperative mortality and morbidity in adult LT patients.^12, 13^

Moreover, to date, no systematic review has evaluated fluid management and blood transfusion during both intraoperative and postoperative periods in paediatric LT recipients.

Therefore, this study aimed to evaluate the effect of perioperative fluid management (fluid replacement and blood transfusion) during the postoperative period in paediatric LT recipients. Our primary outcomes were mortality rate, length of hospital and PICU stay, and number of mechanical ventilation days. Moreover, the secondary outcomes of our study were clinical outcomes such as readmission, the need for postoperative blood transfusion, the occurrence of postoperative complications, and graft health.

## Methods

A systematic review was conducted to identify articles that discussed the outcomes of perioperative fluid management in paediatric LT patients. This review followed the Preferred Reporting Items for Systematic Reviews and Meta-Analyses guidelines.^14^ The PROSPERO registration number for this systematic review is CRD42023423224.

### Eligibility Criteria

The inclusion criteria for this review were (1) studies that addressed fluid management in paediatric (<18 years old) liver transplant recipients during the perioperative and postoperative periods; (2) studies involving perioperative fluid management, including fluid replacement using isotonic and colloid agents, perioperative FB, blood transfusion, and blood component transfusion; (3) randomized controlled trials and observational analytical studies (cohort studies, case studies, cross-sectional studies); (4) studies with the main outcomes of mortality rate, mechanical ventilation days, PICU LOS, and hospital LOS; (5) studies on clinical outcomes such as readmission, the requirement for postoperative blood transfusion, and postoperative complications (AKI, acute lung edema, pneumonia, acidosis); and (6) studies on additional outcomes such as pulmonary complications (pulmonary edema, acute respiratory distress syndrome, pneumonia), graft complications (graft failure, posttransplant cholangiopathy, stricture), cardiovascular complications (arrhythmia, shock, thromboembolic events), hepatic arterial thrombosis, relaparotomy, acidosis, acute lung edema, leakage anastomosis, and coagulopathy.

The exclusion criteria were (1) combined liver-kidney transplantation; (2) death prior to postoperative day 3; (4) liver failure; (5) preoperative liver supportive therapy [Molecular Absorbents Recirculating System (MARS)]; (5) studies assessing surgical techniques; (6) systematic reviews, case reports, or case series; and (7) studies for which the full text was not available.

### Search Strategy

A systematic search was conducted using the following bibliographic electronic databases: PubMed, EBSCOhost, Embase, ProQuest, and Google Scholar, as well as a manual search to identify literature discussing perioperative fluid management in paediatric LT by a medical librarian as a collaborator. The search using electronic databases was performed between 19^th^ May and 21^st^ June 2023. A manual search was performed on 12^th ^December 2023. The search terms used were liver transplant, paediatric, children, fluid management, fluid resuscitation, FB, blood transfusion, mortality rate, and length of hospital stay. In some cases, due to difficulty in retrieving articles regarding the topic, keywords concerning only the population and intervention were used to find as many articles as possible (Supplementary Tables 1-5). The search was limited to 33 years (1990–2023) from the publication date. Excluded studies were not published in English.

### Data Collection and Analysis

Three authors independently analyzed the titles, abstracts and full texts retrieved from the databases. The information extracted from the selected studies was shared among the three authors, and discrepancies were resolved through discussion.

Several data points were extracted from the included studies for analysis:

⦁  Study design and methodology

⦁  Participant demographics, such as age, sex, baseline characteristics, and indications for LT.

⦁  Intervention: type of fluid management, infusion rate/FB, and transfusion dose

⦁  Outcomes: mortality rate, mechanical ventilation days, PICU LOS, hospital LOS, readmission, posttransplant transfusion requirement, postoperative complications, and graft health.

### Risk of Bias Assessment

The strength of evidence of the included studies was assessed using the Joanna Briggs Institute (JBI) critical appraisal tool.^15^ Assessment of the study quality consisted of 11 points and was performed by two independent reviewers. Any discrepancies between the assessment points were discussed with a third- party until a consensus was reached.

## Results

### Study Selection

**Figure 1:**
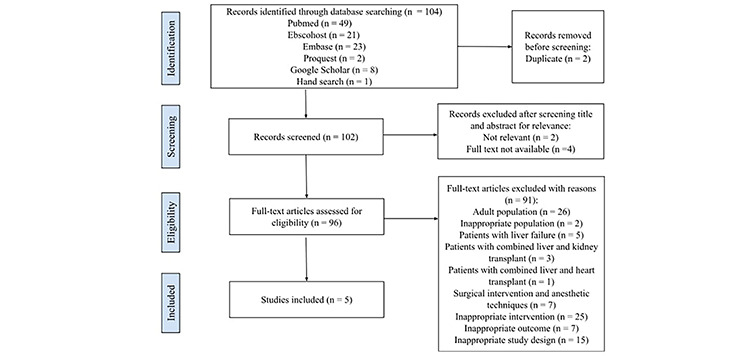
PRISMA flowchart for the selection of included studies

The search strategy involving five electronic databases (PubMed, EBESCOhost, Embase, Proquest, and Google Scholar) yielded 103 potentially relevant records. After eliminating duplicates, 101 records were assessed based on titles and abstracts, resulting in the identification of 95 potentially relevant publications. Through a comprehensive review of the complete texts of the remaining articles and subsequent exclusions for reasons such as inappropriate populations, interventions, outcomes, study designs such as case reports and reviews, and content related to surgical intervention and anaesthetic techniques, five studies were deemed suitable for inclusion in this systematic review. The flowchart in Figure 1 outlines the screening and selection process.

### Study Characteristics

All included studies were retrospective cohort studies. The sample size ranged from 129-333 paediatric LT recipients. We identified three different interventions or factors studied: intraoperative fluid administration (n = 2 studies),^16, 17^ massive transfusion (MT) versus non-massive transfusion (n = 2 studies),^18, 19^ and positive FB within 72 h of surgery (n = 1 study).^6^ The outcomes studied varied greatly and included mortality (n = 2 studies),^16, 18^ risk factors for MT (n = 2 studies),^17, 19^ventilator-free days (n = 1 study),^6^ duration of mechanical ventilation (n = 1),^17^ LOS (n = 3 studies),^6, 17, 19^ PICU LOS (n = 2 studies),^6, 17^ postoperative complications (n = 3 studies),^6, 16, 17^ and graft health (n = 3 studies).^16, 18, 19^ The complete characteristics of the five included studies, including population demographics, details of the fluid therapy intervention (whether it was performed preoperatively or postoperatively), presence of a control group, and outcomes of the studies, are listed in Table 1.

The included studies used various approaches to examine the effects of fluid management and blood transfusions for paediatric liver transplants. Most of the included studies examined risk factors for blood transfusions.^17, 18, 19^ Elevated white blood cell counts, low platelet counts, and the use of cadaveric donors have emerged as robust predictors of MT during paediatric LT.^18^ Moreover, technical graft variants, prolonged operative time, and specific transfusions were identified as risk factors for MT and estimated blood loss.^19^ Notably, patient weight emerged as a significant risk factor for MT, which posed a substantial risk of 30-day graft loss.^19^ A noticeable pattern indicated a tendency for increased volume transfusions in infants, particularly in instances of total parenteral nutrition-related liver failure, as well as in third transplants when compared with second and primary transplants.^17^

### Quality Appraisal Analysis

As determined by the JBI checklist evaluation, most the included studies achieved an “excellent” quality rating, attesting to their robust methodological standards. The quality appraisals of all studies using the JBI tool are listed in Table 2.

### Study Results

In terms of mortality, Dai et al.^16^ reported that 30-day, 90-day, 1-year, and 2-year mortality rates were greater among children receiving lactated ringer’s (LR) solution than among those receiving normal saline (NS) during surgery. Jin et al.^18^ reported that the 6-month mortality rate was not significantly different between the MT group and the non-massive transfusion group (7.3% vs. 7.1%, *P*=0.964).

Two included studies examined risk factors for blood transfusions.^18, 19^ Elevated white blood cell counts, low platelet counts, and the use of cadaveric donors emerged as robust predictors of MT during paediatric LT.^18^ Moreover, technical graft variants, prolonged operative time, and specific transfusions were identified as risk factors for both MT and estimated blood loss.^19^

Two studies evaluated the association between FB and mechanical ventilation. A cumulative FB of >20% in the first 72 h following LT reduced the likelihood of ventilator-free days at 28 days (the number of days patients are free from ventilators and alive within the first 28 days after LT).^6^ Another study focusing only on intraoperative fluid management during paediatric LT concluded that there was an association between intraoperative FB and the duration of postoperative mechanical ventilation in which every 10 mL kg^-1^ h^-1^ of intraoperative fluid administration increased the duration of postoperative ventilation by more than 12 hours.^19^

Three studies assessed the association between blood transfusion and fluid administration and length of hospital stay. Villarreal et al.^19^ reported that MT and massive estimated blood loss were associated with significantly longer lengths of stay (31.5 days in patients receiving a MT compared to 11 days in patients who did not receive a MT). Similarly, Efune et al.^17^ concluded that hospital LOS was independently correlated with total intraoperative blood product administration (sum volume of all blood products administered during surgery). The study revealed that for every 1 mL kg^-1^ of total blood product administered intraoperatively, LOS increased by 0.1 days. Additionally, a study by Winters et al.^6^ demonstrated that a cumulative FB of more than 20% within 72 h following LT operation led to an additional hospital day [adjusted incidence rate ratio: 1.39, 95% confidence interval (CI): 1.10-1.77].

In the context of PICU hospital days, Winters et al.^6^ emphasized the impact of FB and suggested that a FB exceeding 20% at 72 h postoperatively is associated with an increased length of PICU and hospital stay. Another study by Efune et al.^17^ reported a median ICU LOS of 4.3 days (interquartile range: 2.7, 6.8). In addition, the ICU LOS was independently correlated with the intraoperative time of hypotension (r2 = 00318).^17^

Although Winters et al.^6^ did not identify any differences between the groups in terms of the likelihood of postoperative complications. Efune et al.^17^ reported that for every 1 mL kg^-1^ h^-1^ of intraoperative fluid administered, paediatric patients receiving LT had an increased risk of developing either hepatic artery or portal vein thrombosis in the postoperative period (odds ratio: 1.053, 95% CI: 1.001, 1.107). However, they did not find any association between postoperative AKI and intraoperative fluid administration.^17^ Additionally, Dai et al.^16^ reported that AKI occurred within 7 days postoperatively in 6.6% of recipients in the LR group and 4.9% of recipients in the NS group.

In terms of graft health, Jin et al.^18^ examined the association between MT and graft failure. Higher graft failure rates within 6 months were observed in the MT group than in the control group.^18^ However, Villarreal et al.^19^ reported that while MT is not statistically linked to overall graft survival, it does pose a substantial risk for 30-day graft loss, although the result is not statistically significant. Intraoperative fluid management using LR yielded higher incidence rates of early allograft dysfunction (EAD) and primary non-function (PNF) than did the use of NS in paediatric LT patients.^16^ Notably, patient weight emerged as a significant risk factor for MT, which posed a substantial risk of 30-day graft loss.

##  Discussion

Our systematic review included various outcomes of perioperative fluid management strategies for paediatric LT patients. To the best of our knowledge, we are the first to review these outcomes in the paediatric population.

Haemodynamic instability due to surgical procedures and blood loss necessitates intravascular expansion to ensure optimal tissue perfusion. Dai et al.^16^observed increased mortality rates in children receiving RL compared with those receiving NS. Despite not entirely corresponding to physiological conditions, NS is a commonly used crystalloid; however, it may cause hyperchloremic metabolic acidosis or AKI as a side effect. Because of their more physiological composition, LRs are being increasingly used. However, the liver is the primary lactate metabolizer; hence, impaired function in grafts and reperfusion injury in LT may contribute to further increases in lactate concentrations. This increase was associated with graft failure and death in previous studies and hence may be a potential mechanism of increased mortality in RL-receiving patients. Nevertheless, the choice of crystalloid solution may depend on the indication and morbidities of the patient and hence might affect the population studied.^16, 20, 21^

Jin et al.^18^ reported a lack of a significant difference in the 6-month mortality rate between massive and non-massive transfusion groups. This lack of significance may be because of the various factors that may contribute to the mortality rate in LT recipients, such as the preoperative estimated glomerular filtration rate (eGFR) or the occurrence of perioperative complications, including bacterial infection. Another study by Gordon et al.^22^ reported a significant difference in 1-, 5-, and 10-year mortality rates in paediatric patients receiving a high-volume transfusion; however, the definition of MT used differed between the two studies, in that Gordon et al.^22^ defined high-volume transfusion as >27.5 mL kg^-1^. The Gordon et al.^22^ study also revealed that even low-volume transfusions are associated with major postoperative complications. Kloesel et al.^23^ reported no difference in mortality between the massive and nonmassive bleeding groups receiving LT. Although the definition used was similar to that used by Jin et al.,^18^ the follow-up period was only up to 72 h. The varying results of these studies may be due to the various definitions of massive blood loss and transfusion used by the studies and the length of mortality follow-up.

The risk factors for MT that were identified included elevated white blood cell counts, low platelet counts, and the use of cadaveric donors. Children who undergo LT are more likely to be susceptible to bacterial infection because of repeated inflammation of the abdominal cavity and complications of liver failure requiring preoperative invasive procedures. Both of these processes can cause PAs, and their release leads to increased blood loss.^18, 24^ Previous studies have shown similar results, where a low platelet count is associated with MT because of further impairment of coagulation function, which can cause more bleeding.^25, 26^ Cadaveric donors were shown in a previous study to be a risk factor for MT because of the severe diseases these patients had. These donors were mostly used when patients underwent emergency operations or did not have a living donor.^27^ Ulukaya et al.^28^ reported that operative time was an independent risk factor associated with an increased transfusion volume. Kloesel et al.^23^ reported that surgeries lasting >600 min were an independent risk factor for massive bleeding events. Both of these studies confirmed the results found in this review. In addition, an elevated preoperative international normalized ratio, decreased hemoglobin level, and decreased platelet count were risk factors for massive bleeding (which may warrant the increased use of specific blood products) and MT.

A positive FB was associated with longer mechanical ventilation in two of the studies reviewed. Patients with a FB are at a risk of increased fluid accumulation, which results in worsening oxygenation. Complications such as pulmonary edema cause impaired gas exchange, requiring oxygenation and prolonged intubation, and increased susceptibility to bacterial infections. The effect size and, however, remains variable between studies. Prolonged mechanical ventilation was associated with increased mortality and morbidity and consumption of 50% of intensive care unit resources.^17, 29^ The associations between intraoperative FB and the duration of mechanical ventilation were weak, although another previous study by Carrier et al.^30^ reported that restrictive intraoperative fluid management is associated with a shorter duration of mechanical ventilation. The weak correlation observed may be due to the decision to extubate at the discretion of the anaesthesiologist and to the maintenance of open abdomen. The choice of fluid, whether colloid or crystalloid, may also affect the volume of fluid administered because greater volumes of crystalloids than colloids are required to meet targets. Another study by Chang et al.^11^ Reported that patients who received > 260 mL kg^-1^ intraoperative fluid had more days of mechanical ventilation than those who received less fluid. Although not a primary concern for reducing the duration of mechanical ventilation, FB is under the control of the anaesthesiologist; hence, the optimization of outcomes through target-controlled FB is important.^17^ There is still a need for the best monitoring of fluid responsiveness in children.

The studies we reviewed showed that MT and estimated blood loss, increased total intraoperative blood product administration, and a cumulative FB of >20% within 72 h of the operation lead to a longer LOS. The length of ICU stay was also correlated with the same cumulative FB cut-off and hypotension duration during the operation.^6, 17, 18^ This result also agrees with another study of paediatric LT patients, where patients with high intraoperative fluid volumes (>260 mL kg^-1^) were found to have longer hospital LOSs and PICU days than those who received less fluid. These patients were also found to have an increased need for RBC transfusion in the postoperative period.^11^Kloesel et al.^23^ reported that massive bleeding increases the number of PICU days and that massive bleeding may also correspond to increased blood product administration. Another study also confirmed that FB, in addition to cold ischemia time and the cause of liver disease in LT recipients, affects the length of hospital stay.^31^ Furthermore, healthcare is inevitably tied to outcomes relative to cost, where the ultimate goal is to be as efficient as possible.^32^ Modifying the LOS and number of PICU days through controlled fluid management may be an impactful factor for efficiency.

Some reported complications included postoperative thrombosis and AKI. Increased intraoperative fluid administration increased the risk of thrombosis in the hepatic artery or portal vein. Vascular thromboses are caused by the hypercoagulable state of transplant recipients. Strategies employed to avoid thrombosis include a positive FB; however, no guidelines are universally adopted for this type of mitigation.^6^ Interestingly, Winters et al.^6^ reported no association between FB and vascular complications, whereas Efune et al.^17^ reported that increased intraoperative fluid levels increase the risk of developing thrombosis. Similarly, conflicting results were found in other studies. Chang et al.^11^ also did not find any correlation between intraoperative fluid volume and hepatic artery thrombosis (HAT). Another retrospective study noted that paediatric patients with HAT had greater cumulative fluid levels compared with those without HAT events.^33^ Coagulopathy becomes exacerbated in LT patients, especially those with a history of previous operation that consumes coagulation factors; hence, during LT, there may be increased blood loss, which results in increased intraoperative fluid administration.^34^ The conflicting results may be due to previous patient conditions that may affect fluid administration.

Although the quantitative evaluation of fluid administration and AKI did not yield any associations, the evaluation of the type of fluid showed that the occurrence of AKI was greater in patients who received LR than in those who received NS. Postoperative AKI was linked to surgical duration and prolonged cold ischemia time in another study. Haemodynamic instability from significant bleeding may decrease the oxygenation of the kidneys, causing injury.^35^ The incidence of AKI was not significantly different between patients receiving LR and those receiving NS in this study. This was likely because other factors are related to AKI, such as cold ischemia time, which can be reduced by living donor transplantation and explains the low incidence of AKI in this study. Other factors include pre-transplantation eGFR and patient comorbidities, which may affect AKI occurrence.^16^

Two factors are considered to be associated with graft health: MT and the type of intraoperative fluid. MT was reported to be a risk factor for 30-day graft loss, and graft loss tended to increase within 6 months. In terms of the type of fluid administered, patients receiving LR had a greater incidence of EAD and PNF than those receiving NS.^16^ A study using machine learning revealed that elevated sodium levels are a risk factor for graft failure. The hypernatremia observed is hypothesized to reflect the administration of large volumes of pRBCs, fresh frozen plasma (FFP), or albumin and hence can be related to MT during surgery.^36^ Nacoti et al.^37^ also found that the dosages of perioperative transfusion of packed RBCs and FFP were independently correlated with graft survival. The selection of crystalloid solution may contribute to graft health because lactate is mainly metabolized in the liver. LR infusion is associated with increased lactate levels in paediatric LT recipients. However, graft function is also affected by graft quality, thrombotic events, electrolyte imbalances, cold and warm ischemia time, reperfusion injury, and infections.^16, 35^

The studies included in this review had several limitations. First, they were performed on a relatively small sample size, which may have caused the results to be less precise; moreover, they were retrospective cohorts that are prone to selection bias. Second, the studies were conducted in a specific population within reach of their medical centers and hence may not be generalizable to other institutions. Furthermore, one study had an unclear measurement of outcomes and follow-up and was less reliable than the other four included studies. Consequently, there is a need for future research to validate the results found in these studies and further explore the associations suggested because there is still a sparse evidence in the paediatric literature. Prospective research with larger sample sizes and diverse patient populations is required.

## Conclusion

In conclusion, our review highlights the various outcomes of perioperative fluid management strategies in paediatric patients receiving LT in situations where evidence is lacking. Quantitatively and qualitatively, fluid management can impact the mortality rate; the length of mechanical ventilation; the length of hospital and PICU stays; and the incidence of complications such as thrombosis, AKI, and graft dysfunction. These findings underscore the multifaceted impact of perioperative fluid management in this vulnerable patient population, emphasizing the need for better fluid management and evaluation strategies in paediatric LT recipients.

## Supplementary Materials

Supplementary File 1-5 Click here to access: https://d2v96fxpocvxx.cloudfront.net/df15911b-c92e-4fb1-a3c6-b4a159ab0d2e/pdfs/supplement-1.makale.pdf

## Figures and Tables

**Table 1. Characteristics of the Included Studies:** 

**Author**	**Year**	**Country**	**Population**	**Intervention**	**Control**	**Outcomes(s)**	**Results**
**Intraoperative period of the intervention**							
Dai et al.^16^	2021	China	333 paediatric living donor LT recipients	Intraoperative fluid management using the LR	Intraoperative fluid management using the NS	The primary outcome was the mortality rate at 90 days, whereas additional measures encompassed early allograft dysfunction, primary non-function, acute renal injury, and the number of days without hospitalization (days alive after discharge within the first 30 days following liver transplantation)	- The average volumes per body weight were 234±67 and 223±76 mL kg^-1^, respectively, with no significant difference (*P *> 0.05). - The 90-day mortality rate was higher in the group receiving the LR solution than in the NS group (11.5% vs. 0.0%). - The LR group showed higher incidences of early allograft dysfunction (19.7% vs. 3.3%) and primary nonfunction (11.5% vs. 0.0%) than the NS group. - Within 7 days postoperatively, acute renal injury occurred in 6.6% of recipients in the LR Group and 4.9% in the NS group. - Hospital-free days and PICU-free days were not significantly different between the two groups.
Efune et al.^17^	2023	USA	286 paediatric LT recipients	Intraoperative fluid administration using a formula for total fluids = crystalloid (mL) + (5% albumin in mL x 1.5) and indexed it to weight (kg) and duration of anaesthesia (hours)		Duration of mechanical ventilation postoperatively, ICU LOS, hospital LOS, vascular thrombosis (hepatic artery or portal vein), and AKI in the postoperative period	- The median intraoperative fluid administration was 12.5 mL kg^-1^ h^-1^ (IQR: 8.7, 16.9). - The median intraoperative blood product administered was 20.1 mL kg^-1^ (IQR: 8.9, 45.2). - The median duration of postoperative mechanical ventilation was 10.8 h (IQR: 0.0, 35.4). - The median ICU LOS was 4.3 days (IQR: 2.7, 6.8). - The median hospital LOS was 13.6 days (9.8, 21.1). - There was a weak correlation between intraoperative fluid administration and the duration of postoperative mechanical ventilation in paediatric patients receiving LT (r^2^ = 0.037, *P*=0.001). - Hospital LOS was independently correlated with total intraoperative blood product administration (r^2^ = 0.229, *P*=0.001). - For every 1 mL kg^-1^ h^-1^ of intraoperative fluid administered, paediatric patients receiving LT had an increased risk of developing either hepatic artery or portal vein thrombosis in the postoperative period (OR: 1.053, 95% CI: 1.001, 1.107). No association between postoperative AKI and intraoperative fluid administration was observed.
Jin et al.^18^	2016	Korea	249 paediatric LT recipients	Massive intraoperative transfusion	No MT	Risk factors for massive intraoperative transfusion, graft failure rate, and mortality at 6 months	- The overall amount of red blood cell transfusion administered to all patients averaged 126.7±175.4 mL kg^-1^. - Elevated white blood cell count, reduced platelet count, and the use of cadaveric donors were significant predictors of massive transfusion during paediatric liver transplantation. - In the MT group, there was a higher graft failure rate within 6 months than that in the control group (6.6% vs. 1.8%, *P*=0.068). - There was no significant difference in patient mortality rates within 6 months between the intervention and control groups (7.3% vs. 7.1%, *P*=0.964).
Villarreal et al.^19^	2019	USA	250 paediatric LT recipients	MT and massive EBL	No MT or massive EBL	Risk factors contributing to massive intraoperative blood loss/transfusion, LOS, and graft loss in 30 days.	- The median estimated EBL was 9.8 (5.5‐21.5) mL kg^-1^, and the median amount of blood transfused during surgery was 16 (6.9‐28.8) mL kg^-1^. - The average LOS in the groups with MT and massive EBL was significantly longer than that in the groups without (31.5 days vs. 11 days) (*P*=0.001). - Technical graft variants, extended operative time, and transfusion of FFP, platelets, and/or cryoprecipitate were found to be significant independent risk factors for both MT and EBL, whereas admission from home was considered a protective factor. - The weight of the patients was found to be a significant risk factor for MT alone. - Although MT was not statistically linked to overall graft survival, it posed a significant risk of 30-day graft loss.
Postoperative period of intervention (72 hours postop)							
Winters et al.^6^	2022	USA	129 paediatric LT recipients	FB assessment in the first 72 h after surgery	Three groups based on FB (<10%, 10-20%, and >20%)	PICU and hospital length of stay, VFD at 28 days, Day 3 severe kidney injury, and postoperative complications (infections, biliary and vascular complication).	- Thirty-seven patients (28.7%) had a FB of 1020%, and 26 patients (20.2%) had >20% FB. - Having >20% FB was linked to a higher probability of an additional day in the paediatric intensive care unit (aIRR: 1.62, 95% CI: 1.18-2.24), an extra day in the hospital (aIRR: 1.39, 95% CI: 1.10-1.77), and a reduced likelihood of ventilator-free days at 28 days (aIRR: 0.85, 95% CI: 0.74-0.97). - There were no differences between the groups in the likelihood of postoperative complications.

LT, liver transplantation; LR, lactated ringer; NS, normal saline; PICU, paediatric intensive care unit; LOS, length of stay; AKI, acute kidney injury; MT, massive transfusion; EBL, estimated blood loss; VFD, ventilator-free days; FFP, fresh frozen plasma; FB, fluid balance, aIRR, adjusted incidence rate ratio; CI, confidence interval.

**Table 2. Quality Appraisal of the Included Studies:** 

**JBI Checklist**	**Dai et al.^16^**	**Efune et al.^17^**	**Jin et al.^18^**	**Villareal et al.^19^**	**Winters et al.^6^**
						Were the two groups similar and were they recruited from the same population?	Yes	Yes	Yes	Yes	Yes
Were the exposures measured similarly to assign people to both the exposed and unexposed groups?	Yes	Yes	Yes	Yes	Yes
Was the exposure measured in a valid and reliable manner?	Yes	Yes	Yes	Yes	Yes
Were the confounding factors identified?	Yes	Yes	Yes	Yes	Yes
Were strategies to address confounding factors stated?	Yes	Yes	Yes	Yes	Yes
Were the groups/participants free of the outcome at the start of the study (or at the moment of exposure)?	Yes	Yes	Yes	Yes	Yes
Were the outcomes measured in a valid and reliable manner?	Yes	Yes	Yes	UC (graft loss had unclear definition)	Yes
Was the follow-up time reported sufficient to be long enough for outcomes to occur?	Yes	Yes	Yes	Yes	No
Was the follow-up complete, and if not, were the reasons for loss to follow-up described and explored?	Yes	Yes	Yes	UC	Yes
Were strategies to address incomplete follow-up utilized?	Yes	Yes	Yes	Yes	NA
Was appropriate statistical analysis employed?	Yes	Yes	Yes	Yes	Yes
Conclusion regarding the study quality	Excellent	Excellent	Excellent	Good	Excellent

NA, not applicable; UC, unclear; JBI, Joanna Briggs Institute.
